# Transcriptomic and alternative splicing reprogramming by low-dose gamma irradiation in tomato under ToBRFV infection

**DOI:** 10.1016/j.bbrep.2026.102626

**Published:** 2026-05-19

**Authors:** Abozar Ghorbani, Mahsa Rostami, Davoud Koolivand

**Affiliations:** a Department of Plant Protection, Faculty of Agriculture, University of Zanjan, Zanjan, Iran; b Nuclear Agriculture Research School, Nuclear Science and Technology Research Institute (NSTRI), Karaj, Iran

**Keywords:** Gamma irradiation, Alternative splicing, ToBRFV, Transcriptomic reprogramming, Systemic acquired resistance

## Abstract

Tomato brown rugose fruit virus (ToBRFV) is a rapidly spreading pathogen that threatens global tomato production by overcoming established genetic resistance mechanisms. Conventional seed disinfection strategies often fail to eliminate the virus, necessitating the exploration of alternative antiviral approaches. In this study, we examined the effects of low-dose gamma irradiation (15 Gy) on transcriptomic reprogramming and alternative splicing (AS) in tomato seedlings infected with ToBRFV. Through RNA sequencing (RNA-Seq), distinct AS patterns were identified between irradiated and non-irradiated plants, with significant enrichment in exon skipping and alternative splice site usage in the gamma-treated group. High-throughput RNA sequencing data were analyzed using CLC Genomics Workbench, custom Python scripts, and functional enrichment tools including the STRING database and KEGG REST API. These AS events were non-randomly distributed across the genome, with hotspots located in defense-related loci. Six genes were identified that were both differentially expressed and alternatively spliced (DE-ASGs), including kinases, lipases, and auxin response factors, suggesting a dual-layered regulatory response. Functional enrichment analysis revealed that gamma-induced AS genes were significantly involved in plant–pathogen interactions, MAPK signaling, and hormonal response pathways. Furthermore, predicted miRNA–target interactions indicated sly-miR6024 and sly-miR5303 as central regulators of alternatively spliced transcripts. These findings underscore the role of AS as a key component of gamma-induced antiviral defense and point to a synergistic regulatory network involving transcriptional modulation, alternative splicing, and miRNA-mediated control. This study provides novel insights into radiation-induced resistance and transcriptomic plasticity, offering a foundation for developing stress-resilient tomato cultivars.

## Highlights

• 15 Gy gamma irradiation reshapes AS in ToBRFV-infected tomato seedlings.
• Exon skipping & splice site shifts enriched in defense-linked genomic regions.
• DE-ASGs found: kinases, lipases, ARFs imply dual transcriptional-splicing control.
• Gamma-induced AS genes linked to plant–pathogen, MAPK, and hormone pathways.
• sly-miR6024 and sly-miR5303 predicted as master regulators of spliced transcripts.

## 1 Introduction

Tomato (*Solanum lycopersicum* L.) production is increasingly threatened by emerging viral pathogens, notably Tomato brown rugose fruit virus (ToBRFV), a tobamovirus characterized by exceptional stability and seed transmissibility. Since its initial emergence in the Middle East, ToBRFV has rapidly spread across major tomato-producing regions worldwide, overcoming genetic resistance in commercial cultivars and causing extensive yield losses [1,2]. Traditional phytosanitary measures, such as chemical disinfection with sodium hypochlorite (NaOCl), have limited efficacy in curbing seed-borne transmission, particularly when viral particles persist in internal seed tissues [3]. Consequently, alternative, sustainable strategies for virus management are urgently needed. Recent studies have explored alternative physical seed treatments to reduce ToBRFV transmission. For instance, sustainable cold plasma significantly suppressed ToBRFV in tomato seeds [4]. However, it remains unclear how such treatments reshape transcriptomic or splicing-mediated antiviral responses.

Low-dose gamma irradiation has gained attention not only for its efficacy in seed disinfection but also for its potential role as a biostimulant enhancing plant physiological and stress-responsive processes [5]. Previous research indicates that sublethal doses of ionizing radiation (5–20 Gy) may stimulate germination, promote vegetative growth, and bolster stress resilience by modulating hormonal signaling pathways, enhancing antioxidant defenses, and activating systemic resistance mechanisms [6]. Our previous work demonstrated that 15 Gy of gamma irradiation not only significantly reduced ToBRFV replication in tomato seedlings but also improved chlorophyll content and stem development, indicating the activation of systemic acquired resistance (SAR) mechanisms [7]. Despite these promising outcomes, the underlying molecular mechanisms, particularly those governing antiviral resistance triggered by gamma irradiation, remain largely unexplored. A plausible hypothesis is that irradiation prompts extensive transcriptional reprogramming, particularly through alternative splicing (AS), a post-transcriptional mechanism that increases proteomic diversity and enables swift adaptation to environmental stress [8]. AS has been implicated in various plant responses to abiotic and biotic stressors, including salinity, drought, and pathogen attack, where it modulates genes involved in defense signaling, transcription regulation, and secondary metabolism [9]. Moreover, emerging transcriptomic studies in tomatoes have shown that viral infections such as ToBRFV can elicit extensive reconfiguration of gene expression, including AS in genes related to ion transport, protein phosphorylation, and transcription factor activity [10]. In a recent study, cold atmospheric plasma (CAP) treatment in ToBRFV-infected tomato was shown to extensively reprogram both gene expression and alternative splicing, leading to enhanced antiviral defense [11]. Yet, the specific influence of gamma irradiation on AS profiles during viral infection has not been comprehensively investigated. Recent AI-based pipelines for NGS analysis have also improved virus detection and sequence characterization [12].

In this study, RNA-Seq was employed to analyze the global transcriptomic and splicing landscape in tomato seedlings exposed to ToBRFV infection, with or without prior low-dose gamma irradiation. The central hypothesis suggests that gamma exposure induces condition-specific AS events that help suppress the virus and boost host defenses. The study aims to: (i) catalog AS event types and their genomic distribution; (ii) identify differentially expressed, alternatively spliced genes (DE-ASGs); and (iii) explore potential regulatory mechanisms through GO enrichment, KEGG pathway analysis, and miRNA–target predictions. This comprehensive approach offers new insights into the post-transcriptional regulation of antiviral responses and highlights alternative splicing as a vital aspect of gamma-induced systemic resistance.

## 2 Materials and methods

### 2.1 Plant material and gamma irradiation

ToBRFV-infected tomato seeds from the 'SV 3725 TH' variety, collected from plants infected with ToBRFV, were exposed to a low dose of gamma radiation (15 Gy) [7]. This treatment used a Theratron-780 Cobalt-60 (^60Co) source delivering radiation at a rate of 77.35 mGy/min. Appropriate decay corrections were applied to maintain dose precision. Seeds were placed in Petri dishes designed to ensure consistent exposure across samples. A non-irradiated batch of seeds was maintained as a control. All seeds were germinated under controlled greenhouse conditions, with daytime temperatures of 25–30 °C and nighttime temperatures ranging from 16 to 20 °C.

### 2.2 RNA isolation and sequencing library construction

Total RNA was isolated from seedlings at the four-leaf stage using TRIzol™ Reagent (Thermo Fisher Scientific, USA). Each experimental group, irradiated (G15) and non-irradiated controls, was represented by two independent biological replicates. RNA quality was assessed using a NanoDrop spectrophotometer and agarose gel analysis. RNA libraries were constructed using the Illumina TruSeq RNA Sample Prep Kit and sequenced with 150 bp paired-end reads on an Illumina NovaSeq 6000 platform.

### 2.3 RNA-seq data processing and transcript assembly

**Fig. 1:**
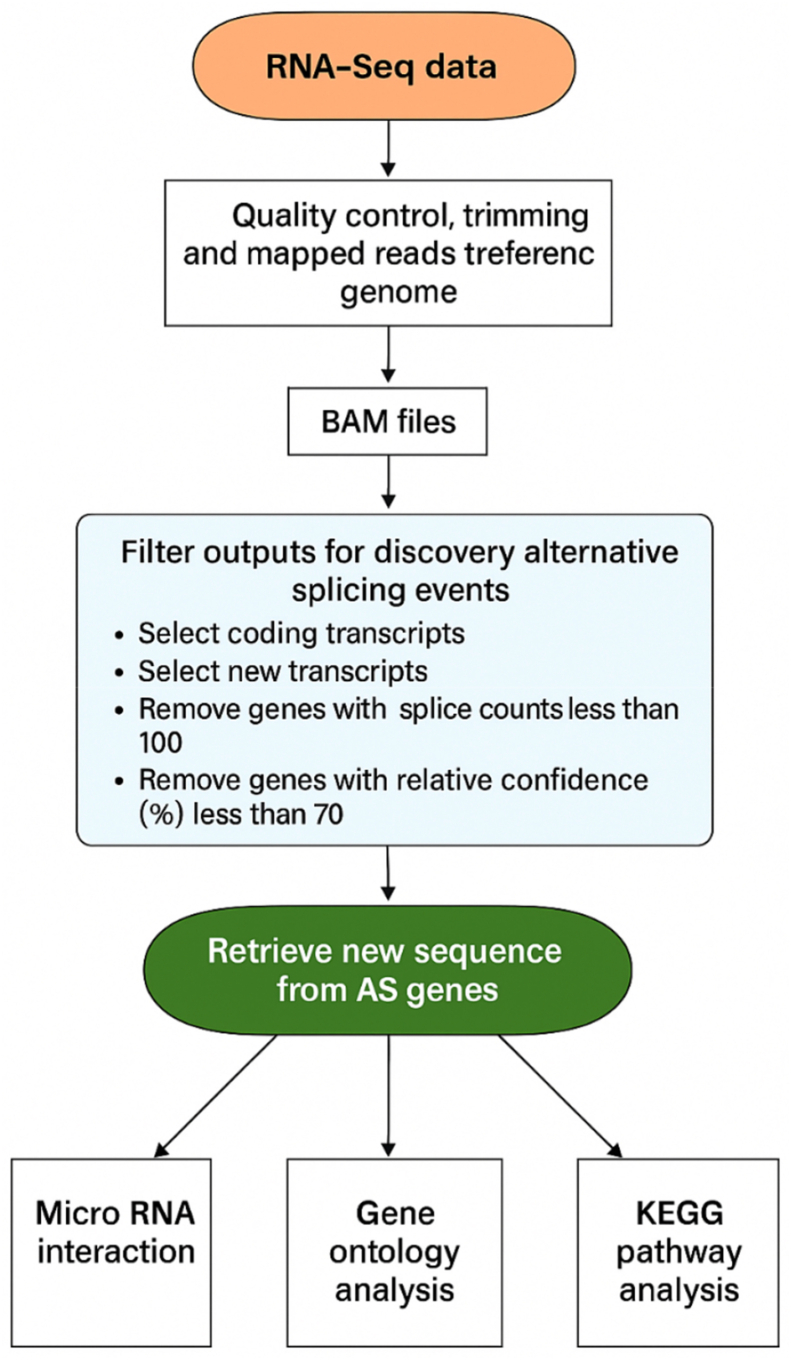
Workflow for the identification and analysis of alternatively spliced genes from RNA-Seq data.

Sequencing reads were processed through CLC Genomics Workbench 25.0 (QIAGEN). Low-quality bases (Q-score <20) were trimmed, and the remaining reads were aligned to the tomato reference genome. The subsequent data analysis for alternative splicing, encompassing preprocessing, event classification, and functional annotation, was carried out as outlined in Fig. 1.

### 2.4 Alternative splicing event analysis

Identification and Comparison of Splicing Events- Custom Python Scripts were designed to analyze AS events exported from the CLC genomic workbench. Data from gamma and control samples were cleaned and organized using pandas into structured DataFrames containing gene IDs, splice site data, chromosomal positions, and splicing evidence. Multi-sheet Excel files were merged to create a unified splicing event table. Comparative analysis flagged AS events as unique to the gamma-treated group, unique to the control, or common to both. A new column labeled "ConditionPresence" classified these categories. Final datasets were saved as Excel files for additional analysis and plotting.

Classification and Visualization of Splicing Types- Using Python, a bar graph was created to categorize AS events based on splice site changes and sequence features. Splicing types included Alternative 5′ splice sites, Alternative 3′ splice sites, and other complex events. A Venn diagram generated with matplotlib_venn illustrated overlapping genes between the treated and control groups. All visualizations were saved in high-resolution TIFF format.

Extraction of Spliced Regions and FASTA Output- A custom Python workflow integrated Biopython and pandas to extract nucleotide sequences of alternatively spliced regions. Genomic coordinates (e.g., “complement (32120.3450)”) were interpreted and used to retrieve sequences from the reference genome. For genes on the negative strand, reverse complements were generated. Output was saved in both Excel and FASTA formats for validation and subsequent analyses (Supplementary Tables 1 and 2).

### 2.5 Differential expression analysis of AS genes

Differential expression analysis was performed using CLC Genomics Workbench. Genes that showed significant expression changes (|Fold Change| ≥ 2, FDR <0.05) and contained unique AS events in the irradiated samples were classified as DE-ASGs.

### 2.6 Functional enrichment and regulatory analysis

Gene Ontology Enrichment Analysis- Gene Ontology (GO) analysis, including Biological Processes (BP), Molecular Functions (MF), and Cellular Components (CC), was performed using the STRING database. Statistically significant enrichment was determined using the Benjamini–Hochberg method, applying a false discovery rate (FDR) cutoff of 0.05.

KEGG Pathway Mapping via Python- An automated Python script was employed for pathway analysis. UniProt IDs were imported and matched to KEGG pathway IDs through the UniProt and KEGG REST APIs. The script included error-handling and retry mechanisms to ensure complete mapping. Frequency summaries of associated pathways were compiled and exported to Excel, with pathway names dynamically retrieved and annotated for contextual interpretation.

Prediction of miRNA Targets- Interactions between *S. lycopersicum* miRNAs and AS genes were predicted using the psRNATarget tool (https://www.zhaolab.org/psRNATarget), referencing miRBase version 22. Only predictions with expectation scores below 3 were retained. Interaction networks were built and visualized in Cytoscape v3.9.1.

## 3 Results and discussion

### 3.1 Alternative splicing event profiling

**Fig. 2:**
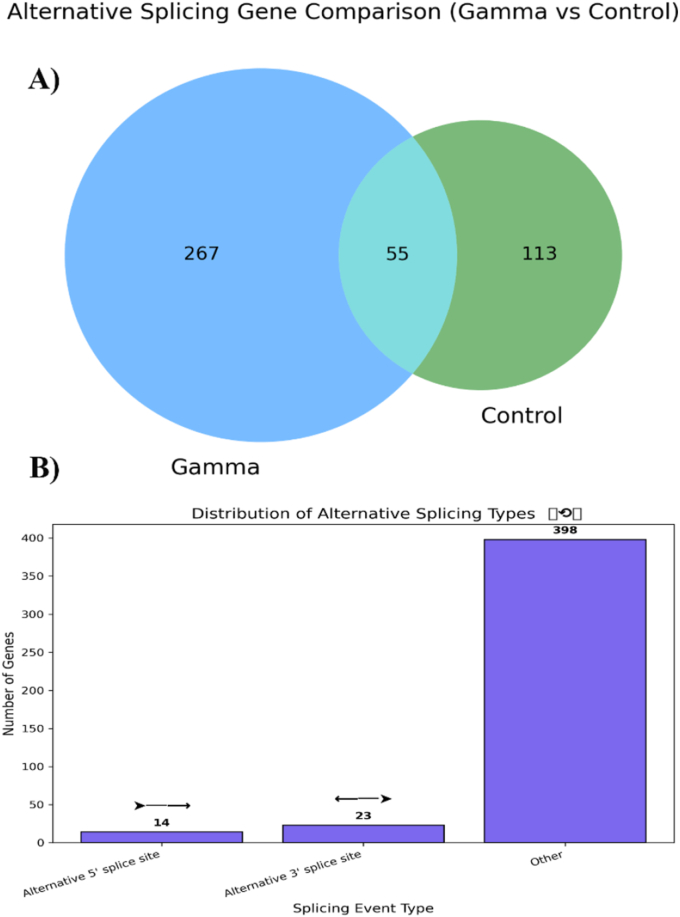
Distribution of alternative splicing (AS) events in gamma-irradiated and control tomato seedlings infected with ToBRFV. Three main AS types were detected: alternative 5′-donor site (A5SS), alternative 3′-acceptor site (A3SS), and other events, including ES, IR, and MXE.

RNA-Seq analysis revealed that gamma irradiation (15 Gy) markedly reshaped the AS landscape in tomato seedlings. In total, 435 genes undergoing AS events were identified across both gamma-treated and control groups. Among these, 267 AS events were unique to the gamma-irradiated seedlings, 113 were specific to the control, and only 55 events were shared between the two groups (Fig. 2A and Additional file 1). These events were detected exclusively in gamma-irradiated plants and represent condition-specific alternative splicing patterns. However, given the reference-guided nature of the analysis, these patterns reflect differential usage of annotated splice sites rather than definitive novel splice variants absent from current genome annotations. These findings indicate that gamma irradiation triggers unique splicing responses beyond those observed under normal conditions. Analysis of the five classical AS types revealed that alternative 5′ splice site (A5SS) and alternative 3′ splice site (A3SS) were present but relatively less abundant, with 14 and 23 events, respectively, while the majority of events fell under the “other” category, which includes exon skipping (ES), intron retention (IR), and mutually exclusive exons (MXE) (Fig. 2B). This distribution is broadly consistent with our previous CAP - induced AS study in ToBRFV-infected tomato, where ES and IR also dominated the splicing landscape [11]. The prevalence of gamma-specific AS events suggests that gamma irradiation influences splicing machinery, enhancing transcriptomic flexibility and enabling the generation of functionally diverse isoforms under potential stress conditions such as viral challenge. Such AS-mediated diversification has been implicated in plant defense responses, particularly in genes involved in pattern-triggered immunity (PTI) and effector-triggered immunity (ETI). In these pathways, IR and ES can produce alternative variants of nucleotide-binding leucine-rich repeat (NLR) receptors and pattern-recognition receptors (PRRs), thereby fine-tuning pathogen recognition and downstream immune signaling [13].

This splicing pattern shift aligns with broader findings across plant species where environmental stress induces transcriptomic plasticity through AS. For example, in *Arabidopsis thaliana*, UV-B radiation has been shown to significantly alter AS patterns, particularly by increasing ES events in stress-responsive genes, thereby modulating their activity at a post-transcriptional level [8,9]. Similar results have been observed in potatoes infected with Potato virus Y, where a significant reprogramming of splice site usage occurred, mainly in genes related to RNA metabolism and transcriptional regulation [14]. Furthermore, low-dose neutron irradiation combined with Tomato yellow leaf curl virus (TYLCV) infection in tomato has been reported to induce splicing changes in stress signaling genes, including kinases and transcription factors [15], which parallels the current findings in tomato, where splicing shifts in irradiated seedlings suggest a regulatory mechanism for fine-tuning gene function during simultaneous irradiation and viral stress. In *Brassica napus* infected with *Leptosphaeria maculans*, AS modifications in key immune and signaling genes have been linked to enhanced tolerance [16]. Reviews on the role of AS in plants emphasize its function as a dynamic regulatory node in metabolic pathways, with ES and IR playing central roles in modulating transcript diversity under stress [17]. Additionally, the transcription factor MdMYB6-like in apples is pivotal in resistance to *Alternaria alternata*, generating isoforms with distinct DNA-binding domains and enabling particular transcriptional responses [18]. In a broader context, AS is recognized not only for increasing transcriptomic diversity but also for contributing to developmental plasticity and immune flexibility, acting as a strategic response to both predictable (e.g., diurnal or seasonal) and unpredictable (e.g., pathogens, radiation) environmental challenges [19]. Overall, these findings underscore that gamma irradiation acts as a potent inducer of AS, similar to other forms of stress, by reprogramming the splicing machinery to rapidly generate isoforms that may enhance physiological robustness.

### 3.2 Chromosomal distribution and gene targets of AS

**Fig. 3:**
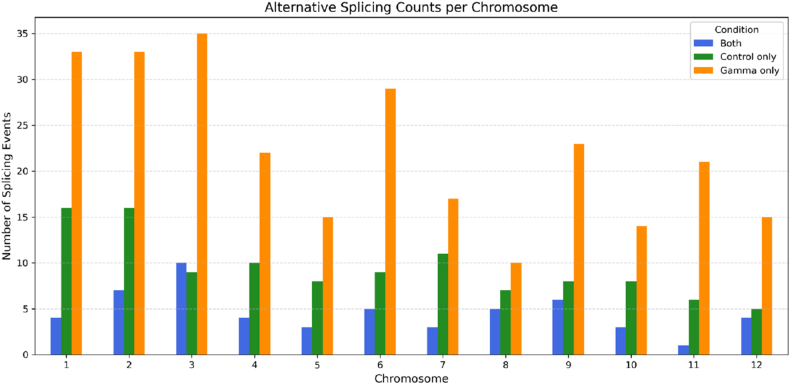
Distribution of types of alternative splicing events in gamma-treated and control seedlings in all chromosomes of tomato seedlings infected with ToBRFV.

Genome-wide analysis of tomato revealed that AS events under gamma irradiation were distributed across all chromosomes, with gamma-specific events consistently exceeding control-specific or shared events. The highest densities of gamma-induced AS were observed on chromosomes 1, 2, and 3, suggesting that these genomic regions may exhibit heightened sensitivity to radiation-induced stress (Fig. 3).

Chromosome 1 harbors a repertoire of transcription factors and metabolic regulators central to stress signaling and secondary metabolism [20]. Chromosome 2 contains the *Tm-1* locus, originally derived from *S. habrochaites*, which confers resistance to ToBRFV when combined with a tolerance locus on chromosome 11. In the resistant ToBRFV-resistant genotype, two distinct copies of *Tm-1* were identified, whereas tolerant and susceptible genotypes carried only a single copy, indicating a potential dosage effect or allelic variation contributing to the resistance phenotype [21]. Chromosome 3 includes loci previously associated with resistance to tobamoviruses in tomato [20], and the observed increase in AS events in this region may reflect post-transcriptional regulation of antiviral defense pathways. Moreover, the observed spatial organization of stress-induced AS events on specific chromosomes in our study aligns with conserved patterns reported across plant species. In soybeans subjected to drought stress, AS events exhibited uniform genomic distribution yet demonstrated significant functional enrichment on chromosomes harboring genes critical for ABA signaling and water stress mitigation [22]. Parallel findings in *Zea mays* under osmotic stress revealed AS event clustering on discrete chromosomes, co-localizing with transcription factor hotspots enriched in WRKY and NAC domain genes [23,24]. Furthermore, integrative analysis of methylome and transcriptome data in maize established that AS peaks frequently coincide with differentially methylated regions genome-wide, reinforcing chromatin state as a key determinant of splicing plasticity during abiotic stress adaptation [25].

### 3.3 Differential expression of alternatively spliced genes

Integrating AS profiles with gene expression data identified six genes in tomato seedlings that were both DE-ASGs in response to 15 Gy gamma irradiation (Table 1). List of genes that were both differentially expressed (FDR <0.05) and showed alternative splicing events in the gamma treatment group. Functional descriptions are based on domain annotation. Positive fold change indicates upregulation; negative values indicate downregulation.

**Table 1:** Differentially expressed alternatively spliced genes in tomato seedling under low-dose gamma treatment, and ToBRFV infection.

Gene	Fold change	FDR p-value	Description
*Solyc01g079600.3*	3.52	0.049891	Lipase_3 domain-containing protein. (730 aa)
*Solyc02g037530.3*	−2.00	0.010329	Auxin response factor; Auxin response factors (ARFs) are transcriptional factors that bind specifically to the DNA sequence 5′-TGTCTC-3′ found in the auxin-responsive promoter elements (AuxREs). (835 aa)
*Solyc04g039730.3*	4.50	6.5E-06	Protein kinase domain-containing protein; Belongs to the protein kinase superfamily. Ser/Thr protein kinase family. (623 aa)
*Solyc04g045620.3*	−2.14	1.09E-05	Uncharacterized protein. (87 aa)
*Solyc09g055590.3*	2.95	0.00033	Uncharacterized protein. (319 aa)
*Solyc12g027850.2*	2.23	0.007215	Protein kinase domain-containing protein; Belongs to the protein kinase superfamily. (467 aa)

Among these genes, *Solyc01g079600.3*, which encodes a lipase domain-containing protein, was upregulated 3.52-fold, and *Solyc04g039730.3*, a serine/threonine protein kinase, showed a significant 4.5-fold increase. Similar regulations of gene expression and AS have been reported in various plant species. In tomato, salt stress was found to induce differential AS in genes, particularly in genes encoding protein kinases, suggesting that salt stress-induced AS supports dynamic signal relay [26]; this observation is consistent with the gamma-induced upregulation of *Solyc04g039730*.*3*, which may facilitate activation of downstream signaling cascades such as MAPKs or calcium-dependent kinases (CDPKs). Similarly, in rice, salt stress was shown to modulate the expression of lipid metabolism genes, potentially affecting membrane stability and stress perception [27]; this supports the gamma-induced upregulation of *Solyc01g079600.3*, which may contribute to lipid remodeling during stress recovery. Besides, in gamma-treated seedlings, several regulatory genes, such as *Solyc02g037530.3* (Auxin response factor), *Solyc04g045620.3*, were downregulated, indicating a possible suppression of specific signaling routes that could help redirect cellular energy toward stress adaptation and immune defense. Energy conservation is a key survival tactic in plants, enabling them to withstand challenging environmental conditions. During stress, plants typically suppress non-essential metabolic activities, channeling available resources toward defense mechanisms and survival processes. Evidence from salt stress studies demonstrates that plants modulate photosynthetic activity and energy metabolism to sustain energy homeostasis and enhance tolerance under adverse conditions [28].

In addition to the genes discussed above, two other DE-ASGs identified in this study further illustrate the extent of transcriptomic and isoform-level regulation induced by gamma irradiation. The gene *Solyc09g055590.3*, which encodes an uncharacterized protein, showed a 2.95-fold increase in expression, suggesting a potential role in stress-responsive regulatory processes that remain to be functionally characterized. Likewise, *Solyc12g027850.2*, a member of the protein kinase superfamily, was upregulated by 2.23-fold and may contribute to phosphorylation-mediated signaling involved in stress perception and downstream defense activation. The presence of both transcriptional modulation and alternative splicing events in these genes supports the idea that low-dose gamma irradiation influences multiple regulatory layers of gene activity. Such coordinated regulation may enable plants to rapidly adjust signaling networks and metabolic pathways in response to viral infection and irradiation-induced stress. Altogether, these findings indicate that gamma irradiation drives a coordinated regulatory mechanism combining transcriptional activation and suppression with AS-mediated isoform reshaping of key functional gene classes, including kinases, hormone regulators, and lipid-modifying enzymes, ultimately enhancing systemic acquired resistance and adaptive resilience. While the present analysis highlights coordinated transcriptional and splicing-level regulation, it is important to note that the resolution of RNA-Seq data at the gene level does not fully capture isoform-specific functional divergence. Future studies incorporating transcript-level quantification and differential transcript usage analyses will be required to precisely determine how specific splice variants contribute to signaling pathway modulation and antiviral defense. Notably, a comparable enrichment of catalytic activity and hormone-related functions was also observed among DE-ASGs in our CAP- treated tomato plants [11], suggesting that distinct physical elicitors may converge on overlapping regulatory modules to enhance antiviral defense.

### 3.4 Functional enrichment: GO and KEGG insights

**Fig. 4:**
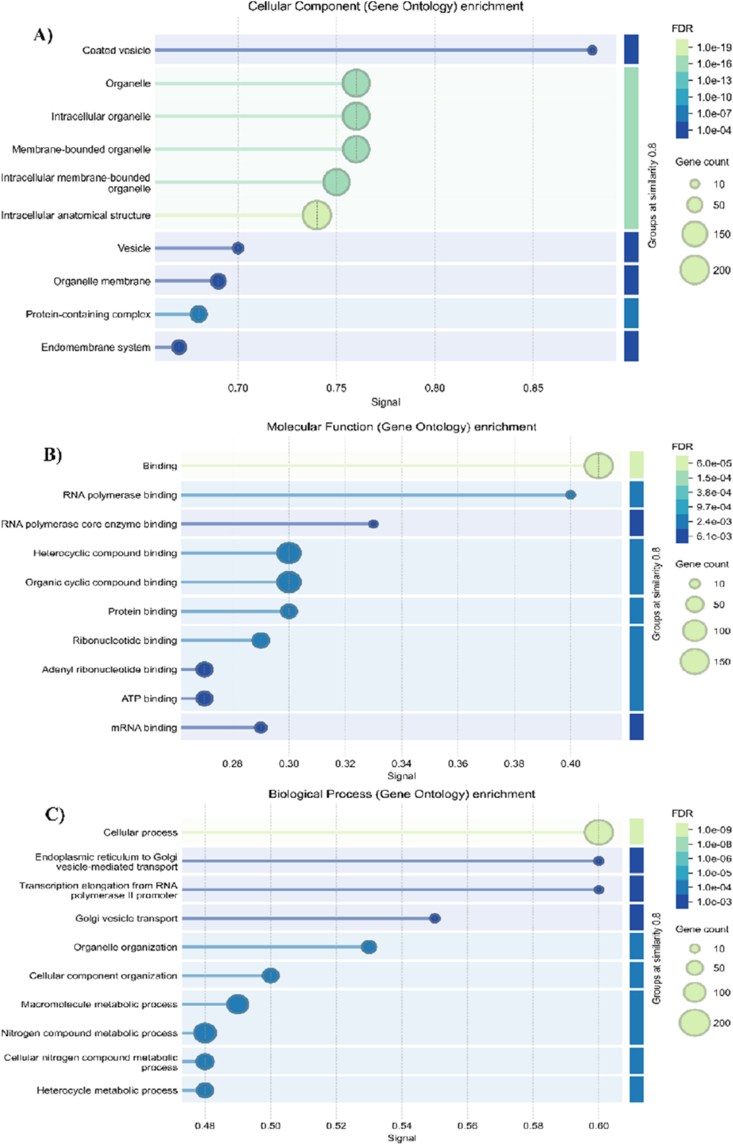
GO enrichment analysis of genes showing gamma-specific alternative splicing events in plant tomatoes infected by ToBRFV. Bar chart of significantly enriched Gene Ontology terms for cellular component (A), molecular function (B), and biological process (C) (FDR <0.05).

To elucidate the functional implications of gamma-induced AS genes, a GO enrichment analysis was conducted across three primary categories—MF, BP, and CC—revealing significant overrepresentation in stress-adaptive functions (Fig. 4).

At the BP level, AS genes were enriched in processes such as endoplasmic reticulum to Golgi vesicle-mediated transport, transcription elongation from RNA polymerase II promoter, Golgi vesicle transport, and organelle organization. This analysis suggests that AS fine-tunes trafficking routes and organelle dynamics in plant cells. For example, genome-wide profiling in *A. thaliana* under salt stress revealed that nearly half of intron-containing genes undergo differential AS, including those involved in vesicle transport, indicating that this mechanism provides an additional layer of stress-responsive regulation [29]. Similar observations in other abiotic stress contexts suggest that AS can rapidly reprogram transport machinery to optimize protein sorting [30]. Within the MF category, there was a strong representation of binding-related terms, including RNA polymerase binding, heterocyclic compound binding, protein binding, and ATP binding. The MF implies that AS modulates protein–protein and protein–nucleic acid interactions, thereby reprogramming transcriptional and metabolic responses. The AS frequently affects transcription factors, RNA-binding proteins, and enzymes, altering their binding specificity and regulatory capacities during plant stress responses [31]. Also, the CC annotations indicated enrichment in coated vesicles, organelles, membrane-bounded organelles, and protein-containing complexes—all compartments relevant to intracellular organization and transport. The CC analysis suggests that AS may determine the subcellular localization and trafficking of plant proteins. Although direct examples of AS-generated isoform localization differences in plants remain limited, the complexity of vesicle transport factors in plants—including COPII and SNARE components—provides a strong mechanistic basis for such regulation [32].

**Fig. 5:**
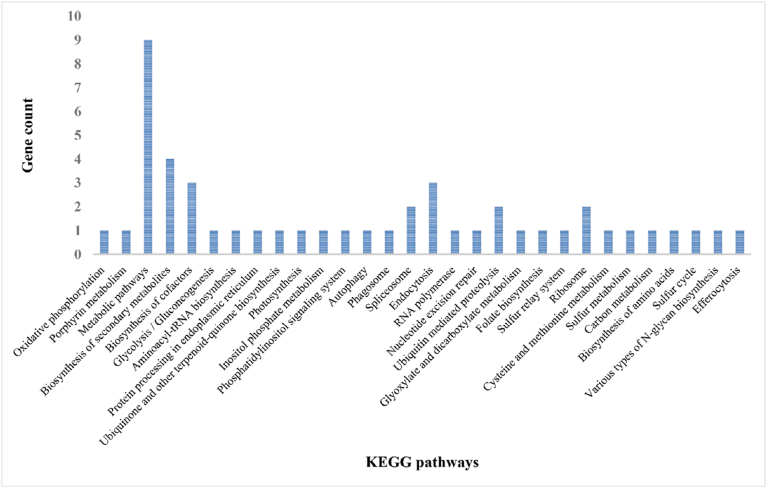
KEGG pathway enrichment of gamma-specific alternatively spliced genes in plant tomatoes infected by ToBRFV.

KEGG pathway enrichment analysis of gamma-induced AS genes revealed involvement in multiple metabolic and signaling pathways (Fig. 5).

The pathways with the highest gene counts were Metabolic pathways (9 genes), followed by Biosynthesis of secondary metabolites (4 genes). The significant enrichment of metabolic and secondary metabolite biosynthesis pathways in AS-related genes suggests that gamma radiation may trigger a reorganization of plant metabolic networks at the post-transcriptional level. Given that alternative splicing directly affects secondary metabolism by modulating the flow of primary metabolites and hormonal homeostasis [33], this mechanism likely enables the plant to fine-tune its metabolite composition, enhancing survival under gamma-induced stress. Moreover, the enrichment of AS genes in protein processing in the endoplasmic reticulum (ER) suggests a potential role for gamma irradiation in modulating protein folding, maturation, and quality control under stress conditions. In plants, ER-related protein processing pathways are critical for managing misfolded proteins generated during environmental stress, thereby maintaining cellular homeostasis [34]. The significant representation of the spliceosome pathway aligns with the central role of AS in regulating stress-responsive transcripts. Similar findings in *Arabidopsis* indicate that stress-induced splicing modulates isoform diversity, enhancing functional plasticity in gene regulation [35]. Furthermore, enrichment in RNA polymerase and nucleotide excision repair pathways highlights the interplay between transcriptional reprogramming and genome stability in response to gamma-induced stress. The detection of the sulfur relay system and metabolic pathway enrichments points to possible adjustments in redox balance and primary metabolism, which are essential for energy supply and signaling during stress adaptation [36]. These enriched pathways—particularly those related to metabolism, secondary metabolite biosynthesis, RNA polymerase, spliceosome, and hormone signal transduction—are largely consistent with our previous transcriptomic and AS study under CAP treatment in ToBRFV-infected tomato, where similar GO and KEGG categories were enriched among plasma-responsive AS genes [11]. This convergence indicates that gamma irradiation and plasma exposure may modulate overlapping post-transcriptional networks that coordinate stress-adaptive and antiviral responses.

Collectively, these enriched pathways suggest that gamma irradiation triggers a coordinated reprogramming of transcription, RNA processing, protein maturation, and metabolic adjustments to optimize plant stress responses.

### 3.5 miRNA-target network analysis

**Fig. 6:**
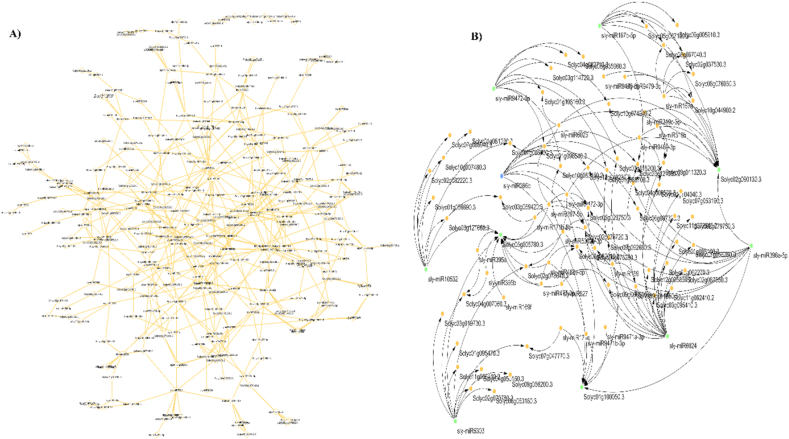
Regulatory network of gamma irradiation-responsive tomato microRNAs and their alternatively spliced target genes under ToBRFV infection, predicted using psRNATarget. A: An interaction network of microRNAs and their alternatively spliced target genes, built with Cytoscape software. B: A subnetwork of hub nodes of the first network, identified using the CytoHubba plugin in Cytoscape.

The miRNAs are small non-coding RNAs that regulate gene expression in plants by guiding transcript cleavage or translational repression. Similar multi-omic network approaches have successfully identified key regulatory modules in plant–pathogen interactions, such as in rice bacterial leaf streak disease [37]. Increasing evidence indicates that their regulatory activity is closely linked with AS, forming a complex system for stress adaptation. In tomato, this interaction between miRNAs and AS is crucial for coordinating developmental and defense responses [38], and similar network-based approaches have identified key regulatory genes in sugar beet defense against curly top virus [39]. In this study, we conducted a systematic analysis of miRNA–ASG interactions in gamma-irradiated tomato seedlings (Fig. 6 and Additional file 2).

In this interaction network, miR6024 emerged as the most prominent hub with the highest number of connections to target genes, indicating its central regulatory role. Previous studies have shown that sly-miR6024 is involved in both plant disease resistance and hormone signal transduction pathways, targeting genes such as AP2-like ethylene-responsive transcription factors, protein phosphatase 2C, and auxin-independent growth promoters [40]. This dual functionality suggests that miR6024 serves as a critical regulatory node, and its extensive interactions with alternatively spliced genes under gamma irradiation may amplify its impact on stress adaptation. Moreover, the analysis identifies sly-miR5303 as a key hub in the miRNA regulatory network of gamma-irradiated tomato seedlings, distinguished by a high level of connectivity at 11. This confirms its role as a master regulator, capable of controlling the expression of many target genes simultaneously. Its central position, along with existing knowledge of its function, underscores sly-miR5303 as a crucial mediator of signaling pathways that coordinate stress response with key developmental processes, especially those related to fruit development and ripening [41].

The importance of specific miRNA families highlights the biological processes they govern, emphasizing their vital roles in plant growth, development, and stress response. Some families stand out as central regulatory hubs, managing the delicate balance between growth, defense mechanisms, and metabolic changes across various conditions. In this study, the miR396 family plays a crucial role, with particularly high significance attributed to sly-miR396a-5p and sly-miR396b. Known as key growth regulators, this family specifically targets Growth-Regulating Factors (GRFs). Its upregulation usually suppresses growth-related genes, redirecting resources toward defense and repair processes, which are typical responses to stress [42]. Additionally, the high connectivity of sly-miR395a and sly-miR395b (each with a degree of 7) highlights their significant role in regulating sulfate assimilation and metabolism. During sulfate deficiency, miR395 acts as a key regulator in both the roots and shoots of *A. thaliana*. Its regulatory role is not limited to *A. thaliana* but is also observed in other plant species, including *Sorghum bicolor*, *B. napus*, and *S. lycopersicum*. This widespread presence indicates a conserved regulatory mechanism across different plant lineages [43]. Furthermore, miR397, particularly sly-miR397-5p (degree = 8), is well recognized for targeting laccase enzymes, which are essential in lignin biosynthesis. Its regulation may contribute to cell wall remodeling as a defense mechanism or influence metabolic pathways during stress conditions [44]. The miR319 family, including sly-miR319b and sly-miR319c-3p, acts as a central hub linking stress responses to critical developmental processes. This connection emphasizes the balance between growth and defense mechanisms [45].

In addition, certain genes are targeted by multiple miRNAs, indicating they are under intensive post-transcriptional regulation. The key gene hubs include *Solyc02g090130*.*3* (degree = 12), Solyc05g005780.3 (degree = 11), and Solyc01g100050.3 (degree = 9). These genes likely play essential roles, and their precise regulation by diverse miRNAs may be a crucial mechanism for fine-tuning the stress response. For example, the gene *Solyc05g005780*, identified as a central node in the network, encodes the gamma-2 subunit of the AP-1 adaptor complex. This complex is crucial for protein sorting in the trans-Golgi network and early endosomes, facilitating the formation of vesicles. Its involvement in intracellular protein transport and vesicle-mediated trafficking is fundamental for stress responses, enabling rapid and targeted reorganization of cellular components and signaling pathways. The presence of a homologous gene in *Arabidopsis* (AT1G60070), crucial for pollen development, underscores its conserved and essential function [46]. Consequently, the regulation of this key gene by multiple miRNAs may provide a sophisticated mechanism for precise control of membrane trafficking and stress adaptation. Notably, a comparable multilayer miRNA–AS regulatory architecture was previously observed in our CAP study conducted under ToBRFV infection. In that work, plasma-responsive alternatively spliced genes were regulated by key stress-associated miRNA families, particularly miR395 and miR319, which are known to modulate metabolic processes, hormone-related signaling, and defense responses. The recurrence of these miRNA families across distinct physical treatments suggests that gamma irradiation and CAP may converge on shared post-transcriptional regulatory frameworks, despite differences in their mode of action [11].

So, this network analysis reveals that a robust and hierarchical miRNA system governs the tomato plant's response to gamma irradiation. Notably, a few master miRNAs, such as sly-miR6024, appear to control extensive sub-networks of target genes, many of which function as critical hubs and undergo alternative splicing. This interaction allows miRNAs to fine-tune the expression of these vital genes, while alternative splicing broadens their functional diversity. From a functional perspective, alternative splicing may influence downstream regulatory outcomes by modulating miRNA accessibility to target transcripts, thereby affecting transcript stability or translational efficiency. In this context, the integration of AS and miRNA regulation observed in this study suggests a coordinated post-transcriptional control mechanism contributing to stress adaptation. However, precise quantification of these downstream effects will require isoform-resolved expression analysis and experimental validation. To confirm their roles as master regulators of radiation stress tolerance, genetic approaches like overexpression or knockdown will be essential. These insights could eventually assist in developing crop varieties with enhanced resilience. However, it should be emphasized that the current network represents predicted regulatory interactions at the gene level. Integration of isoform-specific targeting and experimentally validated interactions would enable a more precise reconstruction of regulatory networks governing gamma radiation-mediated recovery.

## 4 Limitations and future directions

While the present study provides a comprehensive genome-wide characterization of alternative splicing (AS) reprogramming under gamma irradiation and ToBRFV infection, several limitations should be acknowledged. First, the analysis was conducted at the gene level using a reference-guided framework, and therefore did not resolve isoform-specific expression dynamics, differential transcript usage, or potential variation in miRNA binding sites among alternative splice variants. Consequently, the impact of AS on transcript structure—such as gain or loss of regulatory elements—and its precise contribution to post-transcriptional regulation could not be fully delineated.

Second, functional interpretation of AS events was based on enrichment analyses and predicted miRNA–target interactions, without explicit assessment of isoform-specific protein domain architecture. As a result, potential effects of AS on domain composition, including domain loss, truncation, or modification of functional regions, remain to be determined.

Third, experimental validation of predicted splice variants was not performed due to the unavailability of the original biological material. Targeted validation using isoform-specific RT-qPCR and semi-quantitative PCR would be necessary to confirm the presence and relative abundance of individual splice isoforms and to support their functional relevance.

Finally, although the study integrates AS profiling with miRNA interaction networks and functional annotation, a fully resolved regulatory framework linking AS, miRNA activity, signaling pathways, and phenotypic outcomes was beyond the scope of this work. Future studies incorporating transcript-level quantification, isoform-specific target prediction, protein domain analysis, and experimental validation will be essential to elucidate the mechanistic basis of gamma radiation-mediated antiviral responses.

## 5 Conclusion

This research introduces a molecular framework demonstrating how low-dose gamma irradiation reprograms the tomato transcriptome to enhance resistance against the ToBRFV. By employing a multi-omics approach, the study reveals that irradiation triggers a robust, multi-faceted antiviral defense system, predominantly governed by extensive AS reprogramming and tightly regulated by an intricate hierarchical miRNA network. Irradiation prompts significant alterations in the AS landscape, leading to diverse splicing events throughout the genome. Mechanisms such as ES and IR facilitate the precise modulation of defense mechanisms. Furthermore, a vital regulatory layer emerges through a miRNA network, where two master regulators, sly-miR6024 and sly-miR5303, coordinate expansive sub-networks of target genes. These discoveries shed light on radiation-induced bio stimulation, positioning AS as a pivotal driver of antiviral responses. The study emphasizes the potential of low-dose gamma irradiation as an eco-friendly priming strategy and lays a foundation for advanced biotechnological approaches to produce virus-resistant tomato cultivars.

## Consent for publication

Not applicable.

## Ethics approval and consent to participate

Not applicable.

## Availability of data and materials

The authors confirm that the data supporting the findings of this study are available within the article and more data and information on this study are available on request from the corresponding author.

## Funding

Not applicable.

## CRediT authorship contribution statement

A. A.G: conducted laboratory tests and contributed to data interpretation. **Mahsa Rostami:** Software, Validation, Writing – original draft. D.K. revised the manuscript

## Declaration of competing interest

The authors hereby declare that there are no conflicts of interest, financial or otherwise, that could influence the design, execution, or interpretation of this research. No funding sources, commercial entities, or personal relationships have influenced the outcomes or conclusions of this study. All authors have approved this declaration and confirm that the manuscript represents an honest and unbiased account of the research conducted.

## Data Availability

No data was used for the research described in the article.

## Abbreviation

A3SS: Alternative 3′ splice site
A5SS: Alternative 5′ splice site
AS: Alternative Splicing
ASG: Alternatively Spliced Gene
BP: Biological Process (GO enrichment)
CC: Cellular Component (GO enrichment)
CDPK: Calcium-Dependent Protein Kinase
DE-ASG: Differentially Expressed Alternatively Spliced Gene
ER: Endoplasmic Reticulum
ES: Exon Skipping
ETI: Effector-Triggered Immunity
FDR: False Discovery Rate
GO: Gene Ontology
Gy: Gray
IR: Intron Retention
KEGG: Kyoto Encyclopedia of Genes and Genomes
MAPK: Mitogen-Activated Protein Kinase
MF: Molecular Function (GO enrichment)
miRNA: microRNA
MXE: Mutually Exclusive Exons
NaOCl: Sodium Hypochlorite
NLR: Nucleotide-Binding Leucine-Rich Repeat (receptor)
PTI: Pattern-Triggered Immunity
PRR: Pattern-Recognition Receptor
RNA-Seq: RNA Sequencing
SAR: Systemic Acquired Resistance
ToBRFV: Tomato brown rugose fruit virus
TYLCV: Tomato yellow leaf curl virus

## Appendix A Supplementary data

Additional file 1. Genes exhibiting alternative splicing events in tomato plants treated with gamma and infected with ToBRFV. Multimedia component. 1

Additional file 2. Predicted interactions between tomato miRNAs and the alternative splicing gene under gamma treatment. Multimedia component. 2
